# Adiposidade Corporal e Apolipoproteínas em Crianças e Adolescentes: Metanálise de Estudos Prospectivos

**DOI:** 10.36660/abc.20190331

**Published:** 2020-08-19

**Authors:** Gabriela dos Santos de Jesus, Priscila Ribas de Farias Costa, Lucivalda Pereira Magalhães de Oliveira, Valterlinda Alves de Oliveira Queiroz, Carla de Magalhães Cunha, Emile Miranda Pereira, Ana Marlúcia de Oliveira

**Affiliations:** 1 Universidade Federal da Bahia Salvador BA Brasil Universidade Federal da Bahia,Salvador, BA - Brasil

**Keywords:** Criança, Adolescente, Obesidade, Adiposidade, Circunferência da Cintura, Apolipoproteínas, Metanálise

## Abstract

**Fundamento:**

Excesso de adiposidade corporal e doenças cardiovasculares são problemas mundiais com crescente prevalência em crianças e adolescentes, sendo necessário investigar a relação destes, afim de construir estratégias de enfrentamento.

**Objetivo:**

Investigar influência do excesso de adiposidade corporal sobre os níveis séricos de apolipoproteínas B e A1 (ApoB e ApoA1) em crianças e adolescentes.

**Métodos:**

Busca sistemática nas bases de dados Medline/PubMed, Embase, Lilacs, Web of Science, Ovid e Science Direct de coortes consideradas elegíveis, avaliando-se qualidade metodológica e risco de viés; estudos combináveis, com boa qualidade e baixo risco de viés foram analisados com metanálise; a medida sumária utilizada foi a diferença de média ponderada e seu respectivo intervalo de confiança a 95%.

**Resultados:**

8 artigos preencheram os critérios de elegibilidade, incluindo indivíduos com média de idade variando de 9 a 15,7 anos. Para a metanálise, incluíram-se 4 artigos, com total de 7.974 crianças e adolescentes. Observou-se aumento médio de 4,94 mg/dL (IC 95%: 4,22 a 5,67 mg/dL) nos níveis de ApoB naqueles com excesso de adiposidade. Para a ApoA1, identificou-se redução média de -8,13 mg/dL (IC 95%: - 9,09 a -7,17 mg/dL) nos níveis séricos desse marcador em indivíduos com maior adiposidade corporal. Além disso, a influência do excesso de adiposidade corporal sobre os níveis de ApoA1 e ApoB foi maior entre adolescentes do que entre crianças.

**Conclusões:**

O excesso de adiposidade corporal influenciou tanto na redução dos valores de ApoA1 quanto no aumento dos níveis de ApoB em crianças e adolescentes, e tais alterações foram mais relevantes entre adolescentes.(Arq Bras Cardiol. 2020; [online].ahead print, PP.0-0)

## Introdução

A prevalência crescente de excesso de adiposidade corporal em crianças e adolescentes é um problema de saúde em todo o mundo.^1 , 2^ Segundo um editorial da revista The Lancet,^3^ crianças e adolescentes obesos têm maior risco de desenvolver doenças crônicas não transmissíveis (DCNT) na vida adulta, como obesidade, doença cardíaca, diabetes tipo 2, acidente vascular cerebral; além de problemas sociais e psicológicos, como falta de autoestima e estigmatização. Assim, esforços para o enfrentamento da elevada ocorrência do excesso de adiposidade corporal se justificam pela relação com desenvolvimento da doença cardiovascular (DCV), uma das DCNT responsáveis pela alta carga de morbidade em todo o mundo. Os fatores que contribuem para o desenvolvimento de DCV são nomeados fatores de risco cardiometabólicos,^4^ sendo eles excesso de adiposidade, glicemia alta, alterações lipídicas (colesterol LDL e triglicerídeos altos, colesterol HDL baixo), pressão arterial elevada, tabagismo e inatividade física.

O excesso de adipócitos estimula células, citocinas e proteínas pró-inflamatórias a produzirem outras células inflamatórias, interleucina 6 e fator de necrose tumoral alfa, que provocam inflamação e promovem disfunção endotelial. Além desta via, há ação das Apolipoproteínas B (ApoB), que se aderem às células endoteliais, favorecendo maior expressão de moléculas de adesão ao endotélio, e tais efeitos têm resposta sobre formação de placas ateroscleróticas e outros eventos cardiovasculares.^5 - 9^

Evidências indicam que o excesso de adiposidade se correlaciona fortemente com distúrbio lipídicas, como elevação dos níveis plasmáticos de ApoB e redução dos níveis de apolipoproteína A1 (ApoA1).^10 , 11^ Apesar destas evidências bem estabelecidas em adultos, ainda é incipiente tal conhecimento quanto a crianças e adolescentes. Portanto, este estudo teve como objetivo investigar estudos longitudinais que avaliaram a influência do excesso de adiposidade corporal sobre os níveis séricos de ApoB e ApoA1 em crianças e adolescentes.

## Método

### Identificação e Seleção dos Artigos

Trata-se de um estudo de revisão sistemática com metanálise, realizado segundo as diretrizes do *Preferred Reporting Items for Systematic Reviews and Meta-Analyses (PRISMA)* .^12^

### Estratégias de Pesquisa e Critérios de Elegibilidade

Dois investigadores independentes identificaram os artigos nas bases de dados PubMed, Embase, Lilacs, Web of Science, Ovid e Science Direct no período de 16 de dezembro de 2016 a 20 de julho de 2017 utilizando descritores segundo o proposto no Medical Subject Headings (MESH): exposição (adiposidade corporal e termos correlatos: “obesity” OR “overweight” OR “Abdominal obesity” OR “Central obesity” OR “Waist circumference”), e o desfecho (níveis de apoB e ApoA1 e termos correlatos: Apolipoprotein OR ApoB OR “Apo B” OR “Apoprotein B”; ApoA OR “Apo A” OR “Apoprotein A”). Os descritores foram combinados com operadores booleanos “or” e “and” em todas as bases de dados. A definição da estratégia de busca considerou a questão da investigação, estruturada pelo acrônimo População, Exposição, Comparação e “Outcome/Desfecho” (PECO). Somente os termos para os componentes Exposição (E) e Desfecho (O) foram definidos, com a finalidade de evitar especificidade indesejada, restringindo a seleção de estudos.

Os critérios de elegibilidade para a inclusão do artigo no estudo foram: estudos originais conduzidos em humanos, com delineamento observacional prospectivo que envolvessem crianças e adolescentes com idades entre 5 e 19 anos e que analisassem a relação entre adiposidade e níveis de ApoB e ApoA1.

Os estudos deveriam fornecer informações sobre exposição e desfecho, com adoção da média como medida de ocorrência e seu respectivo desvio-padrão. Não foram estabelecidas restrições quanto ao ano, local e idioma de publicação. A seleção dos artigos foi realizada com base nas informações contidas no título e no resumo, adotando-se os critérios de elegibilidade disponíveis em ficha padronizada. Artigos duplicados foram removidos manualmente. Na etapa seguinte, os artigos remanescentes foram lidos na íntegra.

Em caso de discordâncias, um terceiro revisor foi chamado para reunião de consenso. Houve busca manual nas listas de referências dos artigos selecionados com o intuito de identificar possíveis estudos não incluídos na busca eletrônica.

### Critérios de Exclusão

Foram excluídos artigos conduzidos com gestantes, lactantes, indivíduos com DCV, diabetes mellitus, hipertensão arterial e que tivessem se submetido a cirurgia bariátrica. A literatura cinzenta — definida principalmente como resumos de congressos e conferências e *reports* acadêmicos, governamentais e da indústria^14^ — foi incluída apenas pela busca no Ovid. Os autores dos artigos que não informaram a média das concentrações de ApoB e ApoA1 sérica e o desvio-padrão de acordo com a adiposidade foram contatados via endereço eletrônico e, não havendo resposta, foram mantidos na revisão sistemática e excluídos da metanálise.

### Extração de Dados

Dois revisores independentes leram todos os artigos elegíveis na íntegra e registraram em planilha padronizada aqueles que atenderam aos critérios, levando em conta sobrenome do primeiro autor, ano de publicação, tamanho da amostra, média de idade dos participantes, sexo e medida de adiposidade corporal, dados sobre condição de presença ou ausência de excesso de adiposidade ao final do seguimento, média e desvio-padrão de ApoB e ApoA1 séricas dos participantes com e sem excesso de adiposidade ao final do seguimento da coorte.

### Avaliação do Risco de Viés

Dois revisores independentes avaliaram o risco de viés de acordo com a ferramenta *Research Triangle Institute Item Bank* (RTI – Item bank).^15^ Essa ferramenta se organiza em 29 itens, destinados a avaliação de viés em estudos observacionais, dos quais seis deles foram aplicados neste estudo: Q1 – desenho do estudo; Q2 – critérios de inclusão e exclusão explícitos; Q3 – critérios de inclusão e exclusão com medidas válidas e confiáveis; Q5 – igualdade da estratégia para o recrutamento; Q6 – tamanho da amostra; Q7 – nível de detalhe na descrição da exposição; e Q14 – exposições avaliadas usando medidas válidas e confiáveis. Para todas as questões, foram consideradas as respostas 1) sim, 2) não ou 3) não aplicável. Considerou-se alto risco de viés quando o estudo teve dois ou mais pontos negativos ou não aplicáveis; e baixo risco de viés quando apresentou menos de dois pontos negativos ou não aplicáveis.

### Avaliação da Qualidade Metodológica

A qualidade metodológica foi avaliada pelos critérios propostos na escala *Newcastle-Ottawa* ,^16^ que consta de três domínios: 1. Seleção: neste domínio, é identificada a representatividade da amostra, determinação da exposição e ausência de viés de seleção (artigo pode ser pontuado com até quatro estrelas); 2. Comparabilidade entre os grupos: artigo pode ser pontuado com até duas estrelas; 3. Desfecho: análise dos desfechos, comprovação da exposição, avaliação das perdas e adequação do tempo do seguimento (artigo pode ser pontuado com até três estrelas), totalizando nove estrelas. Para este trabalho, foi adotado mínimo de seis estrelas para classificar um artigo com boa qualidade metodológica.^17^

### Análise Estatística

Para a realização da metanálise, foram incluídos quatro estudos. Segundo Higgins & Green,^14^ a metanálise pode ser realizada a partir da combinação de dois ou mais estudos diferentes. Assim, os dados descritivos das variáveis de desfecho segundo a adiposidade corporal ao final do seguimento foram coletadas por dois autores independentes e digitadas em planilha do Excel^®^.

A medida sumária utilizada na metanálise foi a diferença da média ponderada ( *Weighted Mean Difference-WMD* ) da ApoB e da ApoA1 entre indivíduos com e sem excesso de adiposidade corporal, e seus respectivos intervalos de confiança (IC), apresentados em gráfico *forest plot* . Essa medida pode ser usada como estatística sumária em metanálises quando a medida do desfecho em todos os estudos está na mesma escala.^14^

Para calcular a WMD global, utilizaram-se modelos de efeitos aleatórios, apropriados para os estudos com elevada heterogeneidade; tal modelo assume que os estudos tiveram condução diferente, formando amostra aleatória de população hipotética, portanto não há somente um valor que estime a medida de associação, mas uma distribuição de valores.^18 , 19^

Sabe-se que é comum haver heterogeneidade em estudos observacionais e isso influencia a medida de associação. Assim, o pressuposto da homogeneidade foi testado pelo teste Q-Cochran e a magnitude da heterogeneidade foi interpretada pelo percentual de variação entre os estudos considerados, medido com a estatística do I^2^ (teste de inconsistência de Higgins). Um I^2^ menor que 50% foi considerado indicativo de heterogeneidade moderada.^20^ Em caso de alta heterogeneidade (I^2^ maior do que 50 %), utilizou-se a metarregressão.

Considerando que foram incluídos menos de dez estudos na metanálise, não foi possível analisar viés de publicação por meio do teste de Egger e pelo *Funnel plot* . No entanto, a busca abrangente, sensível e sem restrição de idioma e ano contribuiu para diminuir o viés de publicação.

Para todas as análises, adotou-se valor de p menor que 5% como estatisticamente significante. As análises foram realizadas no pacote estatístico Stata for Mac versão 12 ( *Stata Corp, College Station* , TX, USA), usando o comando *metan* para obter a WMD.

## Resultados

### Resultados da Revisão Sistemática

#### Seleção de Estudos

**Figura 1:**
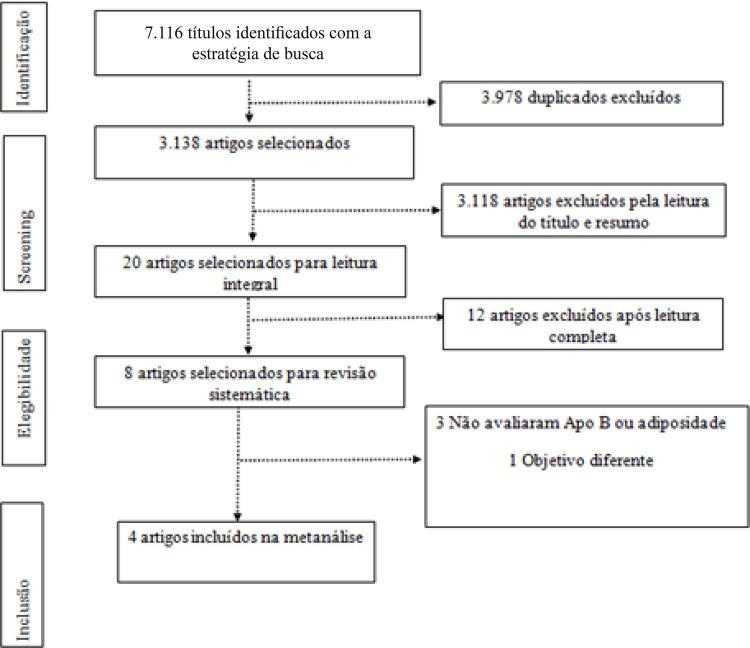
– *Fluxograma revisão sistemática.*

Na busca sistemática, foram identificados 7.116 artigos, dos quais 3.978 eram duplicados. Após leitura do título, resumo e verificação de elegibilidade, foram excluídos 3.118 artigos. Assim, 20 artigos foram selecionados para leitura integral; destes, 12 foram excluídos pelas seguintes razões: não apresentaram dados de ApoB, ApoA1 segundo excesso de adiposidade (três estudos); amostra composta por indivíduos com DCNT (hipertensão, diabetes, síndrome metabólica ou dislipidemia – nove estudos). No total, oito artigos foram eleitos para a revisão sistemática e quatro apresentaram todas as informações sobre a exposição e os desfechos, sendo, portanto, incluídos na metanálise ( Figura 1 ).

#### Características dos Estudos

As principais características dos oito estudos incluídos na revisão sistemática estão descritas na Tabela 1 . Quanto à origem, um estudo foi realizado no Japão, dois na Austrália, um nos Estados Unidos da América, dois na Inglaterra, um na Suécia e um no Canadá, tendo sido publicados entre 2001 e 2016. O tamanho da amostra variou de 59 a 7.589 indivíduos de ambos os sexos, totalizando 15.835 indivíduos, com média de idade de 9 a 15,7 anos. O tempo mínimo de seguimento foi de 12 meses e o máximo de 144 meses, períodos suficientes para a ocorrência do fenômeno.

**Tabela 1:** – Principais características dos estudos incluídos na Revisão Sistemática *Índice de Massa Corporal (IMC); †Densitometria óssea de dupla absorção de raio-X (DXA)

Autor e Ano	Local	Média de idade	Amostra por grupo de exposição		Amostra geral	Seguimento	Medida de adiposidade
			**Com excesso de adiposidade**	**Sem excesso de adiposidade**			
**Falaschetti et al. 2001**	Inglaterra	9,9 anos	1.602	5.987	7.589	120	*IMC
**Larsson et al. 2010**	Suécia	10 anos	29	115	144	120	IMC
**Benson et al. 2012**	EUA	12 anos	87	75	162	12	IMC
**Wilke et al. 2016**	Canadá	11,7 anos	218	412	630	48	IMC e †DXA
**Yamazaki 2008**	Japão	12 anos	19	60	79	144	Adiposidade Rebote
**Bogaert et al. 2003**	Austrália	8,6 anos	-	-	59	12	IMC
**Howe et al. 2010**	Inglaterra	9,9 anos	-	-	7.033	120	DXA
**Mehta et al. 2002**	Austrália	15,7 anos	-	-	139	120	IMC

*Fonte: próprio autor.*

#### Risco de Viés

Os artigos foram avaliados utilizando-se seis questões do RTI, sendo todos os oito artigos considerados de baixo risco de viés. Todos adotaram desenho prospectivo (Q1), apresentaram parcialmente os critérios de inclusão e exclusão (Q2), aferidos com medidas válidas e confiáveis (Q3), com alto nível de detalhe na descrição da exposição (Q7), usando indicadores apropriados e medidas válidas e confiáveis para medi-la (Q14). Dois dos artigos utilizaram a estratégia de recrutamento de participantes entre os grupos (Q5) e, em outros seis artigos, este item não se aplicou por não haver separação por grupos. Somente um artigo21 não declarou os critérios de exclusão (Q2).

#### Avaliação da Qualidade Metodológica

Dentre os oito estudos incluídos na revisão sistemática, todos apresentaram boa qualidade metodológica, alcançando oito^21 - 23^ e sete estrelas.^24 - 28^ A principal limitação observada nos estudos com pontuação sete foi a ausência na descrição dos fatores de controle do estudo no quesito comparabilidade das coortes. Os resultados estão apresentados na Tabela 2 .

**Tabela 2:** – Avaliação da qualidade metodológica dos estudos da revisão sistemática segundo a *Newcastle-Ottawa Scale*

Estudo	Seleção	Comparabilidade das coortes com base no desenho ou análise	Desfecho de cada estudo	Total de estrelas
Benson et al. (2012)	4 estrelas	SP	3 estrelas	7
Bogaert et al. (2003)	4 estrelas	1 estrela	3 estrelas	8
Falaschetti et al. (2001)	4 estrelas	SP	3 estrelas	7
Howe et al. (2010)	4 estrelas	1 estrela	3 estrelas	8
Larsson et al (2010)	4 estrelas	SP	3 estrelas	7
Mehta et al (2002)	4 estrelas	1 estrela	3 estrelas	7
Wilke et al. (2016)	4 estrelas	SP	3 estrelas	7
Yamazaki (2008)	4	SP	3 estrelas	7

#### Resultados da Metanálise

**Figura 2:**
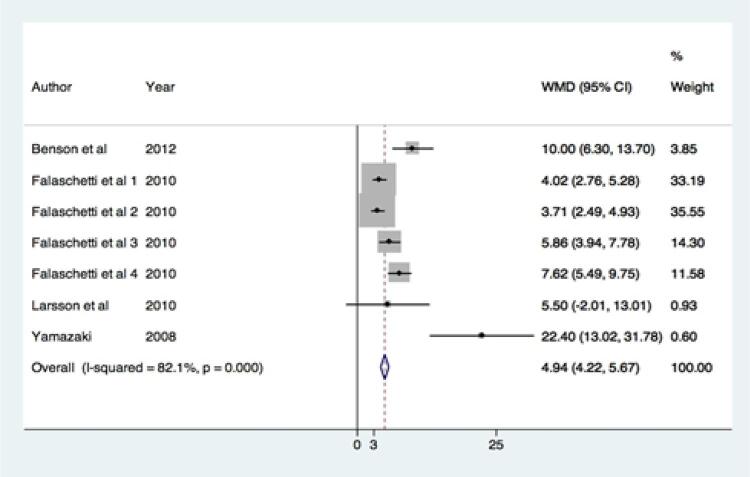
– *Forest plot da influência do excesso de adiposidade corporal sobre a diferença da média ponderada da ApoB em crianças e adolescentes. Falaschetti et al. 1 e 3: correspondem a meninos com sobrepeso e obesidade, respectivamente. Falaschetti et al. 2 e 4: correspondem a meninas com sobrepeso e obesidade, respectivamente.*

Esta metanálise incluiu dados de 7.974 indivíduos, cujos resultados estão apresetados nas Figuras 2 a 5 . Ao avaliar a influência do excesso de adiposidade corporal sobre os valores séricos de ApoB, observou-se aumento médio de 4,94 mg/dL (IC 95%: 4,22 a 5,67 mg/dL) nos níveis deste marcador bioquímico em indivíduos com excesso de adiposidade corporal. Identificou-se redução média de -8,13 mg/dL (IC 95%: – 9,09 a -7,17 mg/dL) nos níveis séricos de ApoA1 em crianças e adolescentes com excesso de adiposidade corporal ( Figuras 2 e 3 ).

**Figura 3:**
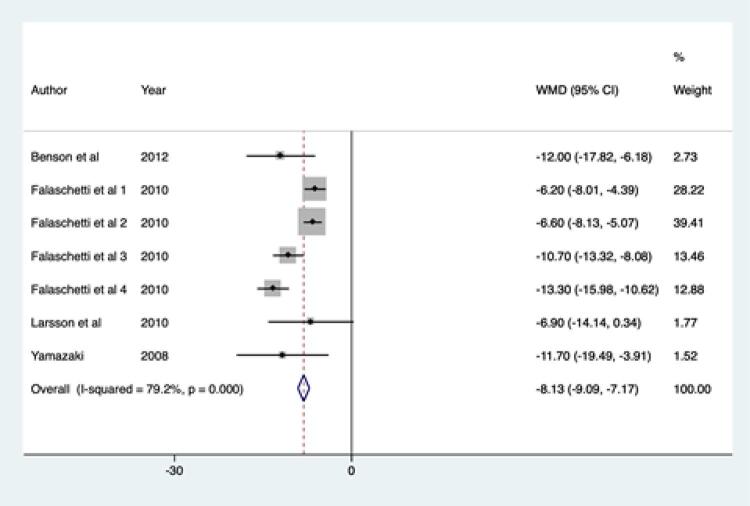
– *Forest plot da influência do excesso de adiposidade corporal sobre a diferença da média ponderada da ApoA1 em crianças e adolescentes. Falaschetti et al. 1 e 3: correspondem a meninos com sobrepeso e obesidade, respectivamente. Falaschetti et al. 2 e 4: correspondem a meninas com sobrepeso e obesidade, respectivamente.*

**Figura 4:**
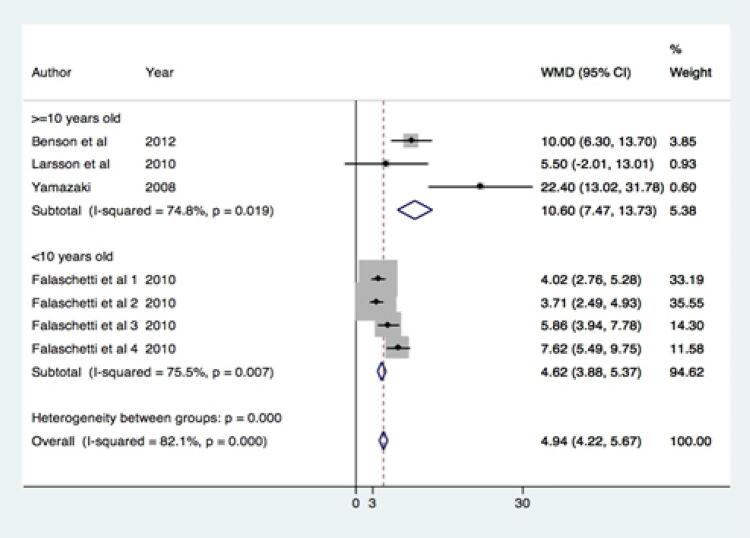
– *Forest plot da influência do excesso de adiposidade corporal sobre a diferença da média ponderada da ApoB, segundo idade de crianças e adolescentes. Falaschetti et al. 1 e 3: correspondem a meninos com sobrepeso e obesidade, respectivamente. Falaschetti et al. 2 e 4: correspondem a meninas com sobrepeso e obesidade, respectivamente.*

Considerando a idade dos indivíduos acompanhados nos estudos originais, foi possível realizar uma análise de subgrupo. Os resultados, descritos na Figura 4 , indicam que o aumento médio nos valores séricos de ApoB em indivíduos com excesso de adiposidade corporal foi maior na população com idade maior ou igual a dez anos (adolescentes), quando comparados com os menores de dez anos (WMD 10,60mg/dL [IC 95%: 7,47 a 13,73] e 4,62mg/dL [IC 95%: 3,88 a 5,37], respectivamente).

**Figura 5:**
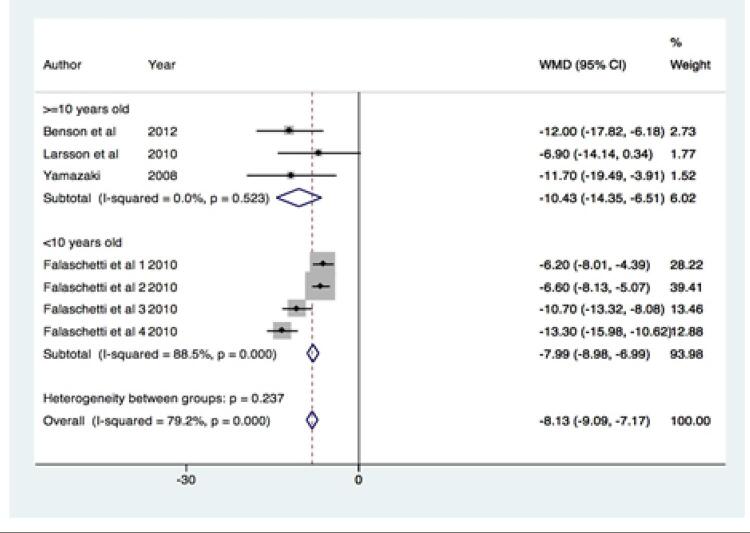
– *Forest plot da influência do excesso de adiposidade corporal sobre a diferença da média ponderada da ApoA1 segundo idade de crianças e adolescentes. Falaschetti et al. 1 e 3: correspondem a meninos com sobrepeso e obesidade, respectivamente. Falaschetti et al. 2 e 4: correspondem a meninas com sobrepeso e obesidade, respectivamente.*

Também para a ApoA1, observou-se que o excesso de adiposidade corporal esteve associado à redução média à maior redução média dos valores séricos deste marcador em adolescentes com idade maior ou igual a dez anos (WMD igual a -10,43mg/dL [IC 95%: -14,35 a -6,51]), comparando-se com menores de dez anos (WMD igual a -7,99mg/dL [IC 95%: -8,98 a -6,99]) ( Figura 5 ).

#### Heterogeneidade e Metarregressão

Os estudos apresentaram alta heterogeneidade, com I2 maior que 50%. As possíveis fontes de heterogeneidade foram investigadas por meio de metarregressão, incluindo as variáveis: sexo (IC 95% -0,76 a 0,71), média de idade (IC 95% -2,06 a 1,71), IMC médio (IC 95% -0,17 a 0,33) e tamanho amostral (IC 95%: 0,12 a 0,36). Nenhuma dessas covariáveis explicou a grade heterogeneidade entre os estudos (dados não apresentados em tabela).

## Discussão

Os resultados indicaram que crianças e adolescentes com excesso de adiposidade têm perfil inadequado dos marcadores ApoB e ApoA1. Identificou-se, ainda, que essas alterações são mais acentuadas em adolescentes do que em crianças. Estas variações nos valores séricos das apolipoproteínas são clinicamente importantes e indicam que crianças e adolescentes com excesso de adiposidade corporal tendem a apresentar perfil inadequado desses marcadores, o que pode predizer maior risco cardiovascular e comorbidades em ciclos mais avançados da vida.

Assim, com base nas evidências disponíveis sobre o assunto e com os resultados desta metanálise, deve-se considerar, para este grupo populacional, a probabilidade de maior risco cardiometabólico em ciclos posteriores da vida. Resultados de alguns estudos têm indicado associação entre excesso de adiposidade corporal e aumento de partículas aterogênicas e diminuição das não aterogênicas em crianças e adolescentes.^29 , 30^ Tem sido registrada, também, média de ApoB mais alta em crianças com sobrepeso e obesidade em comparação a crianças eutróficas.^29^ Neste mesmo estudo,^29^ foi observada correlação positiva entre ApoB, espessura do tecido adiposo epicárdico e triglicerídeos séricos, colocando a ApoB como marcador cardiometabólico com forte correlação com perfil de gordura corporal.

A persistência e agravamento do risco da ApoB com o tempo tem sido observada em adultos jovens identificados com relação IMC/Idade mais acentuada e maior volume de gordura epicárdica. Os autores observaram que esta associação se tornou mais pronunciada 12 anos após a exposição, sugerindo que o risco do excesso de adiposidade tende a permanecer ao longo da vida.^31^

As apoA1 e ApoB são importantes proteínas estruturais e funcionais das partículas de lipoproteínas HDL e VLDL/LDL, respectivamente. Essas proteínas são essenciais para a integridade dessas partículas durante o processamento e para conduzi-las ao seu destino metabólico. Quando há alteração da rota fisiológica, as partículas aterogênicas são direcionadas a órgãos e sistemas, comprometendo suas funções fisiológicas, a exemplo do encaminhamento do colesterol VLDL/LDL para a parede da artéria, levando a comprometimento patológico, como ocorre com a etiologia da aterosclerose. O excesso de adiposidade corporal relaciona-se com elevada concentração sérica de ApoB. Esta partícula é oxidada na parede do vaso, iniciando processo inflamatório com acúmulo local de macrófagos, envolvendo resíduos de LDL e ApoB no espaço subendotelial do vaso, e culminando em disfunção endotelial, formação de ateromas e espessamento da parede vascular.^32 , 33^

Concentrações elevadas de ApoB e LDL estão associadas a aterosclerose e acidente vascular encefálico em adultos.^34^ Evidências recentes indicam que crianças e adolescentes com concentrações elevadas de ApoB e baixas de ApoA1 podem apresentar sinais de aterosclerose em idades mais precoces do que aqueles da mesma idade com concentrações normais desses parâmetros bioquímicos ^35^

Evidências científicas destacam que a exposição a esses fatores de risco nos primeiros ciclos da vida pode contribuir para o desenvolvimento de alterações cardiovasculares em períodos posteriores da vida.^31 , 36^

### Aplicabilidade da Evidência

Sabe-se que as DCV têm grande impacto na morbimortalidade da população, sendo, portanto, eventos que demandam substancial investimento público em assistência à saúde. Ao determinar a relação robusta entre excesso de adiposidade e apolipoproteínas, investigada neste estudo, os resultados podem ter implicações importantes do ponto de vista da formulação de políticas para a prevenção, rastreamento e detecção precoce de sujeitos em risco, favorecendo a construção de medidas de enfrentamento deste problema de saúde.

### Potenciais Vieses no Processo de Revisão

Esta investigação, embora bem desenhada e conduzida, apresenta limitações inerentes aos estudos de metanálise, principalmente no que se refere à ausência de informações, nos estudos primários, sobre as variáveis que permitissem investigar elevada heterogeneidade entre os estudos. Neste estudo, foi investigada a influência das variáveis idade, sexo, IMC médio e tamanho da amostra na metarregressão, uma vez que somente para estas haviam informações disponíveis em todos os estudos. No entanto, sabe-se que a presença de hipotireoidismo pode elevar os níveis de ApoB,^37 , 38^ sendo esta uma variável importante para avaliar possíveis causas da heterogeneidade, porém nenhum dos estudos identificados referiu sobre a presença ou ausência desse distúrbio nos indivíduos avaliados.

O número reduzido de estudos que abordassem este objeto limitou a possibilidade de análise de risco de viés de publicação pelo *funnel plot* . No entanto, alguns autores questionam a real utilidade deste instrumento para esta finalidade, considerando que a interpretação da sua assimetria é subjetiva, podendo haver erros de interpretação sobre o risco de viés de publicação. Soma-se a isso que algumas estimativas de efeito (OR ou diferenças de média) produzidas com o uso do *funnel plot* são naturalmente correlacionadas com seus erros-padrões, podendo produzir assimetria espúria e confusão com viés de publicação.^39^

Muitos estudos identificados tinham desenho transversal, o que não permitiu avaliar o fenômeno de interesse, que é naturalmente longitudinal. Estudos com desenho prospectivo, mas que dosaram ApoB e ApoA1 em um único momento foram incluídos, coma justificativa de que, em crianças menores de nove anos, essas alterações não eram clinicamente importantes,^25^ situação que pode levar a viés de seleção e subnotificação.

### Qualidade da Evidência

Foram incluídas na metanálise evidências de 4 estudos com 7.974 indivíduos acompanhados por 12 a 144 meses, tempo suficiente para ocorrência do fenômeno. Apesar das limitações, esta metanálise foi bem delineada, apoiando-se no uso de ferramentas adequadas para avaliação de risco de viés, análise da qualidade metodológica e realização de análises estatísticas que permitiram investigar possíveis fontes de heterogeneidade.

## Conclusão

Nossos achados sugerem que o excesso de adiposidade corporal influencia na redução dos valores de ApoA1 e aumento dos níveis de ApoB em crianças e adolescentes, sendo estas alterações ainda mais relevantes entre os adolescentes. No entanto, considerando o baixo número de estudos longitudinais identificados nesta metanálise, sugere-se realização de estudos prospectivos que sejam capazes de identificar a influência da adiposidade corporal sobre esses importantes marcadores de risco cardiovascular em adolescentes, considerando seus efeitos negativos ao longo da vida.
