# Supplementary material for: Adiposidade Corporal e Apolipoproteínas em Crianças e Adolescentes: Metanálise de Estudos Prospectivos
Source: Arq Bras Cardiol. 2020 Aug 19;115(2):163–71. [Article in Portuguese] doi: 10.36660/abc.20190331 (PMC8384289; doi:10.36660/abc.20190331)
Supplement: Translation

# Translation: Body Adiposity and Apolipoproteins in Children and Adolescents: A Meta-Analysis of Prospective Studies

**Authors:** Gabriela dos Santos de Jesus, Priscila Ribas de Farias Costa, Lucivalda Pereira Magalhães de Oliveira, Valterlinda Alves de Oliveira Queiroz, Carla de Magalhães Cunha, Emile Miranda Pereira, Ana Marlúcia de Oliveira

**Affiliations:** 1 Brazil Universidade Federal da Bahia, Salvador, BA - Brazil

**Keywords:** Child, Adolescent, Obesity, Overweight, Waist Circumference, Apolipoproteins, Meta-Analysis

## Abstract

**Background:**

Excess Weight and Cardiovascular Diseases are health problems with increasing prevalence among children and adolescents, hence the need to investigate the issues related to them to better deal with the problem.

**Objective:**

To investigate the influence of excess adiposity on the levels of apolipoprotein B and A1 in children and adolescents.

**Methods:**

A systematic search was conducted in the PubMed, Embase, Lilacs, Web of Science, Ovid and Science direct databases, searching for cohort eligible studies and evaluating their results, methodological quality and risk of bias; combinable studies with good quality and low risk of bias were evaluated by meta-analysis. The summary measure used was the weighted mean difference (WMD) with its respective 95% confidence interval.

**Results:**

8 articles attended the eligibility criteria including individuals with age mean varying from 9 to 15.7 years of age. The meta-analysis included 4 articles with a total of 7,974 children and adolescents. It was observed a mean increase of 4,94mg/dL (95%CI: 4,22 to 5,67) in the ApoB levels in individuals with excess of body adiposity. For the ApoA1, we identified a mean reduction of -8,13mg/dL (95%CI: -9,09 to -7,17 mg/dL) in its levels in children and adolescents with higher body adiposity. Beside this, the influence of excess adiposity on the ApoB and ApoA1 levels was higher between adolescents than children.

**Conclusions:**

The excess of body adiposity influenced both the reduction of ApoA1 values and the increase of ApoB levels, being these changes more relevant among adolescents. (Arq Bras Cardiol. 2020; [online].ahead print, PP.0-0)

## Introduction

The growing prevalence of excess body adiposity in children and adolescents is a worldwide health problem.^1 , 2^ According to an editorial published by The Lancet,^3^ obese children and adolescents are at increased risk of developing chronic non-communicable diseases (NCDs) in adulthood, such as obesity, heart diseases, type 2 diabetes, stroke; in addition to social and psychological problems, such as lack of self-esteem and stigmatization. Thus, efforts to face the high occurrence of excess body adiposity are justified by the link with the development of cardiovascular disease (CVD), one of the NCDs responsible for the high burden of morbidity around the globe. The factors that contribute to the development of CVD are called cardiometabolic risk factors,^4^ and include excess adiposity, high blood glucose, lipid changes (LDL cholesterol and high triglycerides, low HDL cholesterol), high blood pressure, smoking and physical inactivity.

Excess of adipocytes stimulates cells, cytokines and pro-inflammatory proteins to produce other inflammatory cells, interleukin 6 and tumor necrosis factor alpha (TNFα), which cause inflammation and promote endothelial dysfunction. There is also the action of Apolipoproteins B (ApoB), which adhere to endothelial cells and favor a greater expression of endothelium adhesion molecules, and such effects have a response on the formation of atherosclerotic plaques and other cardiovascular events.^5 - 9^

Evidence indicates that excess adiposity is strongly correlated with lipid disorders, such as elevated plasma ApoB levels and reduced apolipoprotein A1 (ApoA1).^10 , 11^ Despite this well-established evidence in adults, such knowledge in regard to children and adolescents is still incipient. Therefore, this study aimed to investigate other longitudinal studies that assessed the influence of excess body adiposity on serum levels of ApoB and ApoA1 in children and adolescents.

## Method

### Identification and Selection of Articles

This is a systematic review with meta-analysis, carried out according to the guidelines of the Preferred Reporting Items for Systematic Reviews and Meta-Analyzes (PRISMA).^12^

### Search Strategies and Eligibility Criteria

Two independent researchers searched the articles in the PubMed, Embase, Lilacs, Web of Science, Ovid and Science Direct databases from December 16, 2016 to July 20, 2017 using descriptors as proposed in the Medical Subject Headings (MESH): exposure (body adiposity and related terms: “obesity” OR “overweight” OR “Abdominal obesity” OR “Central obesity” OR “Waist circumference”), and the outcome (ApoB and ApoA1 levels and related terms: Apolipoprotein OR ApoB OR “Apo B” OR “Apoprotein B”; ApoA OR “ApoA” OR “Apoprotein A”). The descriptors were combined with Boolean operators “or” and “and” in all databases. The search strategy considered the orienting question of investigation, structured by the acronym Population, Exposure, Comparison and Outcome (PECO). Only the terms for the Exposure (E) and Outcome (O) components were defined, in order to avoid unwanted specificity, restricting the selection of studies.

The eligibility criteria for inclusion of an article in the study were: original studies conducted with humans, prospective observational design involving children and adolescents aged between 5 and 19 years old and analysis of the relationship between adiposity and ApoB and ApoA1 levels.

The studies were supposed to provide information on exposure and outcome, with the adoption of mean as a measure of occurrence and respective standard deviation. There were no restrictions as to year, place and language of publication. The selection of articles was based on the information displayed in the heading and abstract, adopting the eligibility criteria available in a standardized form. Duplicates were removed manually. In the following step, the articles that were maintained were read in full.

In case of disagreements, a third reviewer was invited for a consensus meeting. We performed manual search in the reference lists of the selected articles to identify possible studies not included in the electronic search.

### Exclusion Criteria

Studies conducted with pregnant women, nursing mothers, individuals with CVD, diabetes mellitus, arterial hypertension and who had undergone bariatric surgery were excluded. The gray literature—defined mainly as abstracts of congresses and conferences, and academic, governmental and industry reports^14^ —was included only in the search on Ovid. The authors of the articles that did not report the mean ApoB and ApoA1 serum levels and the standard deviation according to adiposity were contacted via email and, in case of no response, they were kept in the systematic review and excluded from the meta-analysis.

### Data Extraction

Two independent reviewers read all the eligible articles in full and listed in a standardized spreadsheet the ones meeting the criteria, taking note of the first author’s surname, year of publication, sample size, mean age of participants, sex, and body fat measurement, data on presence or absence of excess adiposity in the end of follow-up, mean and standard deviation of serum ApoB and ApoA1 of participants with and without excess adiposity in the end of follow-up.

### Bias risk Assessment

Two independent reviewers assessed the risk of bias according to the Research Triangle Institute Item Bank (RTI – Item bank).^15^ This tool is organized into 29 items, aimed at assessing bias in observational studies, of which six of them were applied in this study: Q1 – study design; Q2 – explicit inclusion and exclusion criteria; Q3 – inclusion and exclusion criteria with valid and reliable measures; Q5 – equal recruitment strategy; Q6 – sample size; Q7 – level of detail in the description of the exhibition; and Q14 – exposures assessed using valid and reliable measures. For all questions, answers considered were 1) yes, 2) no or 3) not applicable. A high risk of bias was identified when the study had two or more negative or not applicable answers; and low risk of bias when it presented less than two negative points or not applicable.

### Methodological Quality Assessment

Methodological quality was assessed using the criteria proposed in the Newcastle Ottawa^16^ scale, which consists of three domains: 1. Selection: in this domain, sample representativeness, determination of exposure and absence of selection bias are identified (article can be scored with up to four stars); 2. Comparability between groups: articles can be scored with up to two stars; 3. Outcome: analysis of outcomes, evidence of exposure, assessment of losses and adequacy of follow-up time (article can be scored with up to three stars), totaling nine stars. For this work, a minimum of six stars was adopted to classify an article with good methodological quality.^17^

### Statistical Analysis

Four studies were selected to perform the meta-analysis. According to Higgins & Green,^14^ the meta-analysis can be performed by combining two or more different studies. Thus, the descriptive data of the outcome variables according to body adiposity at the end of the follow-up were collected by two independent authors and input in an Excel^®^spreadsheet.

The simple measure used in the meta-analysis was the difference between the weighted mean (Weighted Mean Difference-WMD) of ApoB and ApoA1 between individuals with and without excess body adiposity, and respective confidence intervals (CI), afterwards put in a forest plot graph. This measure can be used as summary statistics in meta-analyses when the outcome measures in all studies are on the same scale.^14^

To calculate the global WMD, random effects models were used, appropriate for studies with high heterogeneity. This model assumes that studies were conducted differently, forming a random sample of hypothetical population, so there is not one value only that estimates the measure of association, but rather a distribution of values.^18 , 19^

It is known that heterogeneity is common in observational studies and this influences the measure of association. Thus, the assumption of homogeneity was tested by the Q-Cochran test and the magnitude of heterogeneity was interpreted by the percentage of variation between the studies considered, measured with I^2^statistic (Higgins inconsistency test). An I^2^less than 50% was considered indicative of moderate heterogeneity.^20^ In case of high heterogeneity (I^2^greater than 50%), meta-regression was used.

Considering that less than ten studies were included in the meta-analysis, it was not possible to analyze publication bias with the Egger test and the Funnel plot. However, the comprehensive, sensitive and unrestricted search based on language and year contributed to reduce publication bias.

For all analyses, a p-value less than 5% was adopted as statistically significant. They were performed in the statistical package Stata for Mac version 12 (Stata Corp, College Station, TX, USA), using the command “metan” to obtain the WMD.

## Results

### Results of the Systematic Review

#### Selection of Studies

In the systematic search, 7,116 articles were identified, of which 3,978 were duplicated. After reading heading and summary and verifying eligibility, 3,118 articles were excluded. Thus, 20 articles were selected for full reading, being 12 excluded for the following reasons: did not present data on ApoB, ApoA1 according to excess adiposity (three studies); sample composed of individuals with CNCD (hypertension, diabetes, metabolic syndrome or dyslipidemia – nine studies). In total, eight articles were chosen for the systematic review. Four had all the information about exposure and outcomes and were, therefore, included in the meta-analysis ( Figure 1 ).

**Figure 1:** – *Flowchart of the systematic review.*

#### Study Characteristics

The main characteristics of the eight studies included in the systematic review are described in Table 1 . As to origin, one study was carried out in Japan, two in Australia, one in the United States of America, two in England, one in Sweden, and one in Canada, having been published between 2001 and 2016. The sample size ranged from 59 to 7,589 individuals of both sexes, totaling 15,835 individuals, with mean age of 9 to 15.7 years. The minimum follow-up time was 12 months and the maximum was 144 months, periods that are sufficient for the phenomenon to occur.

**Table 1:** – Main characteristics of studies included in the systematic review *Body Mass Index (BMI); † Dual-energy X-ray absorptiometry (DXA)

Author and year	Country	Mean age	Sample by exposure group		General sample	Follow-up (months)	Adiposity measure
			**With excess adiposity**	**Without excess adiposity**			
**Falaschetti et al. 2001**	England	9.9 years	1,602	5,987	7,589	120	* BMI
**Larsson et al. 2010**	Sweden	10 years	29	115	144	120	BMI
**Benson et al. 2012**	USA	12 years	87	75	162	12	BMI
**Wilke et al. 2016**	Canada	11.7 years	218	412	630	48	BMI and †DXA
**Yamazaki 2008**	Japan	12 years	19	60	79	144	Adiposity rebound
**Bogaert et al. 2003**	Australia	8.6 years	-	-	59	12	BMI
**Howe et al. 2010**	England	9.9 years	-	-	7.033	120	DXA
**Mehta et al. 2002**	Australia	15.7 years	-	-	139	120	BMI

*Source: author.*

#### Risk of Bias

The articles were evaluated using six questions from RTI, with all eight articles considered to be at low risk of bias. All studies had adopted a prospective design (Q1), partially presented the inclusion and exclusion criteria (Q2), used valid and reliable measures (Q3), had a high level of detail in the description of the exposure (Q7), used appropriate indicators and valid and reliable means of measure (Q14). Two articles used the strategy of recruiting participants between groups (Q5) and, in six other articles, this item was not applied because there was no separation by groups. Only one article21 did not state the exclusion criteria (Q2).

#### Methodological Quality Assessment

Among the eight studies included in the systematic review, all showed good methodological quality, reaching eight^21 - 23^ and seven stars.^24 - 28^ The main limitation observed in studies with a score of seven was the lack of description of control factors in terms of comparability of cohorts. The results are shown in Table 2 .

**Table 2:** – Evaluation of methodological quality of the studies included in the systematic review according to the Newcastle Ottawa Scale

Study	Selection	Comparability of cohorts based on design or analysis	Outcome of each study	Total stars
Benson et al. (2012)	4 stars	SP	3 stars	7
Bogaert et al. (2003)	4 stars	1 star	3 stars	8
Falaschetti et al. (2001)	4 stars	SP	3 stars	7
Howe et al. (2010)	4 stars	1 star	3 stars	8
Larsson et al (2010)	4 stars	SP	3 stars	7
Mehta et al (2002)	4 stars	1 star	3 stars	7
Wilke et al. (2016)	4 stars	SP	3 stars	7
Yamazaki (2008)	4 stars	SP	3 stars	7

#### Results of the Meta-analysis

This meta-analysis included data from 7,974 individuals, whose outcomes are shown in Figures 2 to 5 . When assessing the influence of excess body adiposity on serum ApoB values, an average increase of 4.94 mg/dL was observed (95%CI: 4.22 to 5.67 mg/dL) at the levels of this biochemical marker in individuals with excess body adiposity. An average reduction of -8.13 mg/dL (95%CI: -9.09 to -7.17 mg/dL) was identified in the serum levels of ApoA1 in children and adolescents with excess body adiposity ( Figures 2 and 3 ).

**Figure 2:** – *Forest plot of the influence of excess body adiposity on the difference of weighted mean of ApoB in children and adolescents. Falaschetti et al. 1 and 3: correspond to overweight and obese boys, respectively. Falaschetti et al. 2 and 4: correspond to overweight and obese girls, respectively.*

**Figure 3:** – *Forest plot of the influence of excess body adiposity on the difference of weighted mean of ApoA1 in children and adolescents. Falaschetti et al. 1 and 3: correspond to overweight and obese boys, respectively. Falaschetti et al. 2 and 4: correspond to overweight and obese girls, respectively.*

Considering the age of the individuals followed up in the original studies, we could carry out a subgroup analysis. According to the results, described in Figure 4 , the mean increase in serum ApoB values in individuals with excess body adiposity was greater in the population aged ten years or older (adolescents), when compared to those younger than ten years (WMD 10.60 mg/dL [95%CI: 7.47 to 13.73] and 4.62 mg/dL [95%CI: 3.88 to 5.37], respectively).

**Figure 4:** – *Forest plot of the influence of excess body adiposity on the difference from the weighted mean of ApoB, according to the age of children and adolescents. Falaschetti et al. 1 and 3: correspond to overweight and obese boys, respectively. Falaschetti et al. 2 and 4: correspond to overweight and obese girls, respectively.*

Also for ApoA1, excess body adiposity was associated with a greater mean reduction in the serum values of this marker in adolescents aged ten years or older (WMD -10.43 mg/dL [95%CI: -14.35 to -6.51]), compared to children under ten years old (WMD -7.99 mg/dL [95%CI: -8.98 to -6.99]) ( Figure 5 ) .

**Figure 5:** – *Forest plot of the influence of excess body adiposity on the difference of the weighted mean of ApoA1 according to the age of children and adolescents. Falaschetti et al. 1 and 3: correspond to overweight and obese boys, respectively. Falaschetti et al. 2 and 4: correspond to overweight and obese girls, respectively.*

#### Heterogeneity and Meta-regression

The studies had high heterogeneity, with an I^2^greater than 50%. Possible sources of heterogeneity were investigated through meta-regression, including the variables: gender (95%CI -0.76 to 0.71), mean age (95%CI -2.06 to 1.71), mean BMI (95%CI -0.17 to 0.33) and sample size (95%CI: 0.12 to 0.36). None of these covariables could explain the wide heterogeneity between studies (data not shown in table).

## Discussion

The results indicated that children and adolescents with excess body adiposity have an inadequate profile of ApoB and ApoA1 markers. These changes were also found to be more pronounced in adolescents than in children. These variations in serum values of apolipoproteins are clinically important and indicate that children and adolescents with excess body adiposity tend to present an inadequate profile of such markers, which can predict higher cardiovascular risk and comorbidities in future life cycles.

Thus, based on the available evidence on the subject and the results of this meta-analysis, the likelihood of a higher cardiometabolic risk in later life cycles should be considered for this population group. Results of some studies have indicated an association between excess body adiposity and increase in atherogenic particles or decrease in non-atherogenic particles in children and adolescents.^29 , 30^ There has also been a higher mean of ApoB in overweight and obese children compared to eutrophic children.^29^ This same study^29^ found a positive correlation between ApoB, thickness of epicardial adipose tissue and serum triglycerides, placing ApoB as a cardiometabolic marker with a strong correlation with body fat profile.

The persistence and worsening of the risk of ApoB over time has been observed in young adults identified with a more pronounced BMI/Age ratio and greater volume of epicardial fat. The authors observed that this association became more pronounced 12 years after exposure, suggesting that the risk of excess adiposity tends to remain throughout life.^31^

ApoA1 and ApoB are important structural and functional proteins of the HDL and VLDL/LDL lipoprotein particles, respectively. These proteins are essential for the integrity of particles during processing and to lead them to their metabolic destination. When there is a change in the physiological route, the atherogenic particles are directed to organs and systems, compromising their physiological functions, such as the forwarding of VLDL/LDL cholesterol to the artery wall, leading to pathological impairment, as occurs with the etiology of atherosclerosis. Excessive body adiposity is related to a high serum concentration of ApoB. This particle is oxidized on the vessel wall, giving raise to an inflammatory process with local accumulation of macrophages, involving LDL and ApoB residues in the subendothelial space of the vessel, and culminating in endothelial dysfunction, atheroma formation and thickening of the vascular wall.^32 , 33^

High concentrations of ApoB and LDL are associated with atherosclerosis and stroke in adults.^34^ Recent evidence indicates that children and adolescents with high ApoB and low ApoA1 concentrations may show signs of atherosclerosis at earlier ages than those of the same age with normal concentrations of these biochemical parameters.^35^

Scientific evidence highlights that exposure to these risk factors in the first cycles of life can contribute to the development of cardiovascular changes in later periods of life.^31 , 36^

### Applicability of Evidence

It is known that CVDs have a great impact on the population’s morbidity and mortality, and therefore require substantial public investment in health care. When determining the robust relationship between excess adiposity and apolipoproteins—investigated in this study—, the results may have important implications from the point of view of formulating policies for the prevention, screening and early detection of subjects at risk, favoring the construction of measures to cope with this health problem.

### Potential Biases in the Review Process

Although this investigation was well designed and conducted, it has limitations inherent to meta-analysis studies, especially with regard to the lack of information, in primary studies, on the variables that would allow investigating high heterogeneity between studies. In this study, the influence of the variables age, sex, mean BMI and sample size on the meta-regression was investigated, since only for these there was information available in all studies. It is known that the presence of hypothyroidism can raise ApoB levels,^37 , 38^ an important variable to assess possible causes of heterogeneity; however, none of the studies included in our analysis referred to the presence or absence of this disorder in the individuals evaluated.

The small number of studies addressing this object limited the possibility of analyzing the risk of publication bias by funnel plot. However, some authors question the real usefulness of this instrument for this purpose, considering that the interpretation of its asymmetry is subjective, and there may be errors of interpretation regarding the risk of publication bias. In addition, some effect estimates (OR or mean differences) produced using the funnel plot are naturally correlated with their standard errors, which can produce a false asymmetry and confusion with publication bias.^39^

Many studies had a cross-sectional design, which did not allow the phenomenon of interest to be evaluated, which is naturally longitudinal. Studies with a prospective design, but which measured ApoB and ApoA1 in a single moment, were included under the justification that, in children under nine years old, these changes were not clinically important,^25^ a situation that can lead to selection and underreporting bias.

### Quality of Evidence

Evidence from 4 studies with 7,974 individuals followed for 12 to 144 months was included in the meta-analysis, enough time for the phenomenon to occur. Despite the limitations, this meta-analysis was well designed, based on the use of appropriate tools to assess the risk of bias, analyzing methodological quality and conducting statistical analyses that allowed investigating possible sources of heterogeneity.

## Conclusion

Our findings suggest that excess body adiposity influences the reduction of ApoA1 values and increase in levels of ApoB in children and adolescents, these changes being even more relevant among adolescents. However, considering the low number of longitudinal studies found in this meta-analysis, we suggest further prospective studies that can identify the influence of body adiposity on these important markers of cardiovascular risk in adolescents, considering their negative effects throughout life.
